# Plasminogen Activator Inhibitor-2 Polymorphism Associates with Recurrent Coronary Event Risk in Patients with High HDL and C-Reactive Protein Levels

**DOI:** 10.1371/journal.pone.0068920

**Published:** 2013-07-09

**Authors:** James P. Corsetti, Peter Salzman, Dan Ryan, Arthur J. Moss, Wojciech Zareba, Charles E. Sparks

**Affiliations:** 1 Department of Pathology and Laboratory Medicine, University of Rochester School of Medicine and Dentistry, Rochester, New York, United States of America; 2 Department of Biostatistics and Computational Biology, University of Rochester School of Medicine and Dentistry, Rochester, New York, United States of America; 3 Department of Medicine, Cardiology Unit, University of Rochester School of Medicine and Dentistry, Rochester, New York, United States of America; The Ohio State Unversity, United States of America

## Abstract

The objective of this work was to investigate whether fibrinolysis plays a role in establishing recurrent coronary event risk in a previously identified group of postinfarction patients. This group of patients was defined as having concurrently high levels of high-density lipoprotein cholesterol (HDL-C) and C-reactive protein (CRP) and was previously demonstrated to be at high-risk for recurrent coronary events. Potential risk associations of a genetic polymorphism of plasminogen activator inhibitor-2 (PAI-2) were probed as well as potential modulatory effects on such risk of a polymorphism of low-density lipoprotein receptor related protein (LRP-1), a scavenger receptor known to be involved in fibrinolysis in the context of cellular internalization of plasminogen activator/plansminogen activator inhibitor complexes. To this end, Cox multivariable modeling was performed as a function of genetic polymorphisms of PAI-2 (*SERPINB*, rs6095) and LRP-1 (LRP1, rs1800156) as well as a set of clinical parameters, blood biomarkers, and genetic polymorphisms previously demonstrated to be significantly and independently associated with risk in the study population including cholesteryl ester transfer protein (CETP, rs708272), p22phox (*CYBA*, rs4673), and thrombospondin-4 (*THBS4*, rs1866389). Risk association was demonstrated for the reference allele of the PAI-2 polymorphism (hazard ratio 0.41 per allele, 95% CI 0.20-0.84, p=0.014) along with continued significant risk associations for the p22phox and thrombospondin-4 polymorphisms. Additionally, further analysis revealed interaction of the LRP-1 and PAI-2 polymorphisms in generating differential risk that was illustrated using Kaplan-Meier survival analysis. We conclude from the study that fibrinolysis likely plays a role in establishing recurrent coronary risk in postinfarction patients with concurrently high levels of HDL-C and CRP as manifested by differential effects on risk by polymorphisms of several genes linked to key actions involved in the fibrinolytic process.

## Introduction

Inflammation is widely believed to play a major role in the development of atherosclerosis [1,2]. Evidence is accumulating that one of the processes promoted by inflammation and contributing to atherogenesis is the dysfunctional transformation of high-density lipoprotein (HDL) particles. This transformation is thought to result in a change in HDL from anti-atherogenic to pro-atherogenic [1,3-8]. We have investigated this notion in terms of both incident [9,10] as well as recurrent [11] cardiovascular disease (CVD) risk in population studies that have demonstrated high-risk for patients with concurrently high levels of HDL cholesterol (HDL-C) and C-reactive protein (CRP). To investigate potential pathways leading to increased risk in such individuals, we have assessed associations of risk with functional genetic polymorphisms related to multiple processes involved in development of atherosclerosis. These have included polymorphisms of genes involved in lipoprotein metabolism (*CETP*, *LPL*) [10,11], oxidative stress (*CYBA*) [12], and thrombogenesis (*THBS4*) [12]. We sought to extend these studies by investigating the role of fibrinolysis in the establishment of risk in patient populations having concurrently high levels of HDL-C and CRP. 

Fibrinolysis, the break-down of fibrin clot, is an important process in maintaining vascular homeostasis. Thus, it is not unexpected that fibrinolysis is thought to play a significant role influencing the development of atherosclerosis [13,14]. Fibrinolysis is primarily mediated by plasmin which is generated by activation of plasminogen by plasminogen activators (tissue-type plasminogen activator (tPA) and urokinase-type plasminogen activator (uPA)). Plasminogen activator inhibitor-1 (PAI-1) and plasminogen activator inhibitor-2 (PAI-2) are regulatory proteins involved in the control of fibrinolysis. Inhibition of fibrinolysis derives from covalent binding of PAI-1 and PAI-2 with tPA and uPA resulting in blockage of activation of plasminogen to plasmin. Whereas association of CVD risk with PAI-1 has been well-established [14-16]; for PAI-2, there are few studies addressing this issue [17]. However, with particular regard to the issue of inflammation, potential association of PAI-2 with CVD risk in this setting is suggested by results from a recent study indicating that PAI-2 can induce apoptosis in endothelial cells in the setting of inflammation through inhibitory effects on proteosome function [18].

Human PAI-2 is a 47 kDa non-glycosylated single chain protein of 415 amino acids that has a predominantly intracellular location; although a small fraction is also known to be secreted in a non-traditional secretory pathway as a 60 kDa glycosylated protein [19]. In this regard, it should be noted that in some cases secretion of glycosylated PAI-2 can be significant [20]. Production is mainly by monocytes and macrophages but also by eosinophils, keratinocytes, and microglia. It has also been well-established that a variety of agents including growth factors, hormones, cytokines, vasoactive peptides, toxins, and tumor promoters can upregulate PAI-2 expression to a remarkable degree, in some cases, resulting in increases greater than 1,000-fold [20]. Pro-inflammatory mediators can also upregulate PAI-2 expression significantly [17]. This and the fact that, unlike PAI-1, PAI-2 is resistant to oxidative degradation have led to the notion that near inflammatory foci, PAI-2 may be the primary inhibitor of plasminogen activation [17]. It should also be noted that endothelial cells express PAI-2 and its expression is highly inducible by pro-inflammatory mediators [21].

As noted above, PAI-2 exhibits a predominantly intracellular distribution. Recent efforts directed toward characterization of intracellular functions of PAI-2 suggest potential roles in regulation of apoptosis, cell differentiation, the innate immune response, cell signaling, and neuroprotection [18,20]. Conversely, the predominantly intracellular location of PAI-2 could call into question functionality with regard to inhibition of uPA and tPA. However, a potentially significant role for PAI-2 in the inhibition of fibrinolysis has been proposed based on the notion of liberation of large amounts of intracellular PAI-2 by damaged cells in inflammatory foci [21-23].

Inhibition of fibrinolysis by PAIs involves formation on cell surfaces of covalent complexes of PAIs with uPA or tPA. Next comes internalization of complexes via specific members of the LDL receptor (LDLR) family of endocytosis receptors (LRP-1 and VLDLR) [20,21]. For PAI-1, complexes bind strongly to LDLRs via the PAI-1 portion of complexes resulting not only in internalization but also in cell signaling events. In contrast, for PAI-2, complexes bind to LDLRs via the PA portion of complexes but with less strong binding such that although internalization still occurs, cell signaling events do not. Differential actions resulting from such differences have been proposed to underlie recently reported non-traditional functionalities of PAI-1 and to allow for PAI-2 a more targeted approach for inhibition of plasminogen activation without concomitant cell signaling events [20,24].

To extend our studies investigating factors potentially involved in the establishment of CVD risk as related to HDL in the setting of inflammation, we assessed a potential role for fibrinolysis in this regard by probing a previously characterized subgroup of postinfarction patients defined by concurrently high levels of HDL-C and CRP [11,12] using a genetic polymorphism of PAI-2 (*SERPINB*, rs6095) [25,26]. This seemed like a potentially informative approach especially given the previously cited study demonstrating endothelial cell apoptosis induced by PAI-2 under inflammatory conditions [18]. Further, given the role of the LDLR family of receptors as related to PAI action as noted above, we assessed a polymorphism of LRP-1 (LRP1, rs1800156) [26] for effects on PAI-2 polymorphism-associated recurrent risk.

## Materials and Methods

### Study Population

A subgroup of postinfarction patients from the Thrombogenic Factors and Recurrent Coronary Events (THROMBO) postinfarction study [27] previously identified to be at high-risk for recurrent coronary events comprised the study population [11,12]. The study group (N=166) was a subgroup of non-diabetic THROMBO patients (N=767) defined by concurrently high levels of HDL-C and CRP identified using outcome event mapping, a graphical exploratory data analysis tool [28,29]. This approach allows identification of high-risk patient subgroups as a function of two potential risk parameters (biomarkers) by generation of 3-dimensional scatter plots of risk versus biomarker levels to which a surface smoothing algorithm is applied. High-risk subgroups are manifested as peaks in the mappings.

The original THROMBO prospective postinfarction study [27] investigated a set of blood biomarkers as potential predictors of risk for recurrent coronary events. Patients were enrolled in the study after presenting with a myocardial infarction (MI) (16.8% of enrolled patients already had had at least one MI previous to the presenting MI while for the other 83.2%, the presenting MI was their first MI). Patients were then followed for recurrent coronary events (cardiac death, myocardial infarction, and unstable angina) for a mean period of 26 months. The THROMBO study was carried out with approval of and according to guidelines of Research Subjects Review Boards of participating institutions including acquisition of informed consent.

### Blood Markers

Fasting venous blood specimens were drawn two months after presentation MI. The following seventeen blood biomarkers were determined as described previously [11]: apolipoprotein B (apoB), total cholesterol, lipoprotein-associated phospholipase A_2_ (Lp-PLA_2_), apolipoprotein A-I (apoA-I), HDL-C, triglyceride, glucose, insulin, lipoprotein(a) (Lp(a)), plasminogen activator inhibitor-1 (PAI-1), von Willibrand factor antigen (vWF), fibrinogen, D-dimer, factor VII, and factor VIIa, CRP, and serum amyloid A (SAA).

### Genotyping

Genotyping was performed as described previously [12,28,30,31] for: PAI-2 (*SERPINB*, rs6095), LRP-1 (LRP1, rs1800156), cholesteryl ester transfer protein (CETP, rs708272), p22phox (*CYBA*, rs4673), and thrombospondin-4 (TSP-4) (*THBS4*, rs1866389). All were in Hardy-Weinberg equilibrium except for p22phox (*CYBA*, rs4673). Deviations from Hardy-Weinberg equilibrium were most likely associated with the highly selected nature of the postinfarction study population.

### Statistical Analyses

Statistica 10.0 (StatSoft, Inc., Tulsa, OK) was used for all analyses with significance determined at the p<0.05 level. Comparisons between and among groups were assessed using Mann-Whitney U and Kruskal-Wallis tests, respectively. The distribution of time to recurrent coronary event was estimated using the Kaplan Meier estimator. Cox proportional hazard regression was used to model time to recurrence as a function of clinical covariates, biomarkers and genetic polymorphisms. For these analyses, clinical variables (gender, race, prior MI, ejection fraction ≤ 30 (EF30)) were treated as binary variables; smoking was treated as a tri-level variable (0 - never, 1 - quit, and 2 - current); age, BMI, and blood biomarkers were treated as continuous variables; and genetic polymorphisms were treated as tri-level variables (0 - reference allele homozygotes, 1 - heterozygotes, and 2 - variant allele homozygotes). Cox modeling was performed in two steps. The initial step was a stepwise selection approach using all of the above variables as described and including only those cases with complete data for all variables. The final step was an all-effect model including only those variables found to be significant in the initial stepwise model and again including only those cases with complete data for the significant variables. The final step was performed to take advantage of the fact that, in general, the initial stepwise approach will generate a model with many fewer independent variables than were initially entered. This will then increase the number of cases with complete data allowing for higher statistical power.

Outcome event mapping is a graphical exploratory data analysis tool that can be used to identify high-risk patient subgroups [28,29]. Briefly, 3-dimensional scatter plots of outcome events (z-axis) over a bivariate risk domain (x-y plane) of two continuous biomarker variables are first generated. Coding for outcome is 0 for no outcome event and 1 for an outcome event. Biomarker variables are then transformed to ranks resulting in a more even distribution of patients over the bivariate risk domain. Next, a smoothing algorithm is applied resulting in a surface (outcome event map) with height over the bivariate plane taken as a measure of the outcome event rate. Peaks in mappings correspond to high-risk subgroups. High-risk subgroup patients are identified as those patients contained within the footprint of a region demarcated by a contour line of constant risk the value of which is often taken as the mean outcome event rate in the total population.

## Results

### PAI-2 Polymorphism and Inflammation in the Parent Study Population

**Figure 1:**
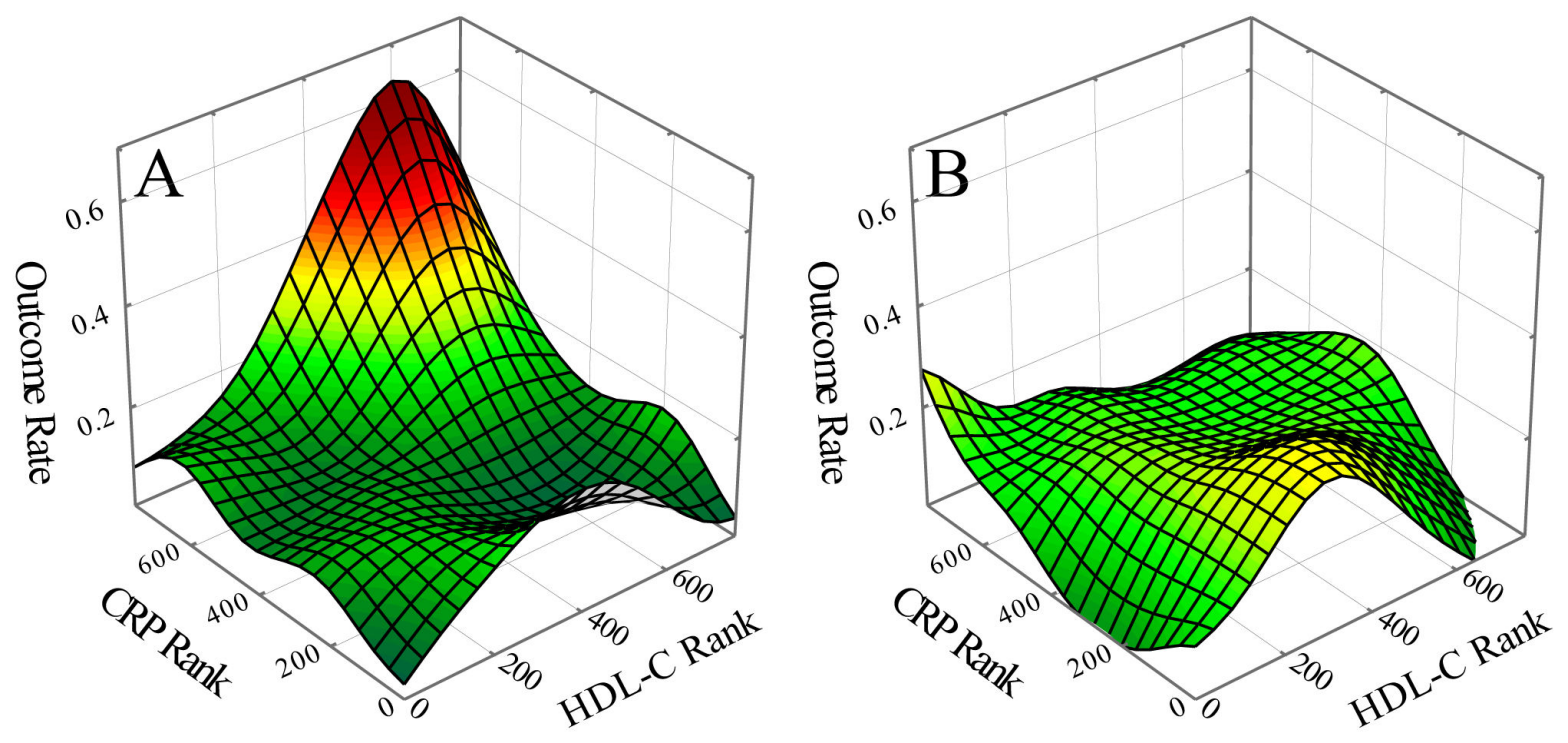
Outcome event mappings demonstrating estimated risk rates over the HDL-C/CRP bivariate domain as a function of PAI-2 polymorphism (*SERPINB*, rs6095) variants. Outcome event mapping for: (A) reference allele homozygotes, and (B) variant allele carriers of the PAI-2 polymorphism.

Characterization of the parent study population of postinfarction patients (N=767) was reported previously [12]. As a preliminary investigation, potential effects of inflammation and PAI-2 polymorphism risk associations in the parent study population were explored. CVD outcome events over time were assessed using univariate Cox regression as a function of each of the seventeen blood biomarkers (see Methods section) in patients having CRP levels greater than the median level of CRP. Results were significant only for HDL-C with increasing levels associated with risk (hazard ratio - 1.02, p=0.035). In view of this result, the parent study population was divided into quadrants based on CRP (2.2 mg/L) and HDL-C (0.96 mmol/L) median levels. Outcome event rates were then assessed as a function of the three PAI-2 polymorphism variants in the four patient quadrants. Results demonstrated significant difference only in the high HDL-C/high CRP quadrant with highest risk associated with reference allele homozygotes (Table 1). This relationship was then further explored using outcome-event mapping to demonstrate estimated risk over the HDL-C/CRP bivariate domain for PAI-2 polymorphism reference allele homozygotes (Figure 1A) versus PAI-2 polymorphism variant allele carriers (Figure 1B). The high-risk peak in the plot of Figure 1A suggests, consistent with the quadrant analysis above, focused reference allele-associated risk for patients with concurrently high levels of HDL-C and CRP; and it is interesting to note that the peak of Figure 1A essentially corresponds to the high-risk peak previously identified using outcome event mapping in patients with concurrently high levels of HDL-C and CRP without reference to genetic polymorphisms [11]. In view of this correspondence, all subsequent analyses were performed on this previously identified and well-characterized patient subgroup.

**Table 1:** Recurrent coronary event rates (%) and number of patients (N) as a function of PAI-2 polymorphism variants for patient quadrants resulting from stratification about CRP and HDL-C median values.

Quadrant	Reference allele homozygote	Heterozygote	Variant allele homozygote	p-values*
Low HDL-C/Low CRP				
Event Rate (%)	14.8	21.0	6.3	0.31
Number of patients	88	62	16	
Patients (%)	53.0	37.3	9.6	

High HDL-C/Low CRP				
Event Rate (%)	7.6	14.0	21.4	0.21
Number of patients	92	57	14	
Patients (%)	56.4	35.0	8.6	

High HDL-C/High CRP				
Event Rate (%)	31.3	12.7	13.3	0.019
Number of patients	83	63	15	
Patients (%)	51.6	39.1	9.3	

Low HDL-C/High CRP				
Event Rate (%)	10.8	17.0	11.8	0.55
Number of patients	93	53	17	
Patients (%)	57.1	32.5	10.4	

* Kruskal-Wallis test.

### High HDL-C/High CRP Patients

Characterization of the high HDL-C/high CRP study population (N=166) in terms of clinical and biomarker parameters have been reported previously [11,12]. Briefly, patients were on average 58.4 ± 11.1 years old, 56% male, 67.5% white, and overweight with BMI of 27.7 ± 5.95 kg/m^2^. Table 2 gives mean values and SDs for continuous variables, percentages of the indicated feature for categorical variables, and smoking score as a function of absence or presence of recurrent coronary events. There were no statistically significant differences for any of the parameters.

**Table 2:** Clinical parameters and blood biomarkers as a function of absence or presence of outcome events.

Parameters	Without Outcomes N=127	With Outcomes* N=39	p-values†
Clinical Covariates			
Age (years)	58.6 ± 11.4	58.1 ± 10.1	0.68
Gender (% Male)	56.7	53.8	0.76
Race (% White)	66.9	69.2	0.79
Prior MI (%)	14.3	13.5	0.91
EF30 (%)	12.4	14.3	0.77
Smoking Score‡	1.04	1.21	0.27
BMI (kg/m^2^)	28.14 ± 6.23	26.41 ± 4.81	0.09

Blood Biomarkers			
ApoB (g/L)	1.23 ± 0.27	1.30 ± 0.33	0.52
Chol (mmol/L)	5.25 ± 1.02	5.59 ± 1.4	0.29
Lp-PLA**2** (μmol/min/mL)	23.35 ± 5.04	25.30 ± 7.47	0.23
ApoA1 (g/L)	1.28 ± 0.25	1.34 ± 0.29	0.38
HDL-C (mmol/L)	1.20 ± 0.26	1.25 ± 0.37	0.54
Trig (mmol/L)	1.98 ± 1.15	2.08 ± 1.19	0.52
Glucose (mmol/L)	5.08 ± 1.44	4.9 ± 1.25	0.44
Insulin (pmol/L)	127 ± 153	108 ± 95	0.36
PAI-1 (µg/L)	27.1 ± 21.1	24.7 ± 20.9	0.34
Lp(a) (mmol/L	0.65 ± 0.61	0.78 ± 0.57	0.09
CRP (mg/L)	9.22 ± 10.7	8.86 ± 5.24	0.14
VWF (%)	168 ± 78	160 ± 79	0.52
Fibr (g/L)	3.89 ± 0.88	3.91 ± 0.93	0.97
D-dim (µg/L)	570 ± 502	537 ± 314	0.45
SAA (mg/dL)	3.13 ± 12.29	1.11 ± 1.22	0.61
FVII (%)	112 ± 55	115 ± 47	0.64
FVIIa (µg/L)	2.88 ± 2.23	2.83 ± 2.29	0.88

* Number of patients with outcome events were as follows: unstable angina - 28, MI - 9, and CHD death - 2

† Mann–Whitney U test.

‡ Smoking score: 0 - never, 1 - quit, and 2 - current.

### Genotypes

Table 3 gives recurrent coronary event rates, patient numbers and percentages for the PAI-2 (rs6095), and the LRP-1 (rs1800156) polymorphisms in terms of reference allele homozygotes, heterozygotes, and variant allele homozygotes. For the PAI-2 polymorphism, there was a monotonic downward trend in event rates in going from reference allele homozygotes to variant allele homozygotes. For the LRP-1 polymorphism, the event rate for the variant allele homozygotes trended higher than that for reference allele carriers. Also included in Table 3 are corresponding results from a previous report assessing recurrent risk for three other polymorphisms in the high-risk subgroup: p22phox (rs4673), thrombospondin-4 (rs1866389), and CETP (rs708272) [12].

**Table 3:** Number of patients, percentage of patients, and recurrent coronary event rate (%) as a function of polymorphisms in terms of reference allele homozygotes, heterozygotes, and variant allele homozygotes.

Polymorphism	Reference allele homozygote	Heterozygote	Variant allele homozygote
PAI-2 (*SERPINB*, rs6095)			
Event Rate (%)	34.7	14.6	6.7
Number of patients	72	48	15
Number of patients (%)	53.3	35.6	11.1

LRP-1 (LRP1, rs1800156)			
Event Rate (%)	21.1	24.5	33.3
Number of patients	57	53	18
Number of patients (%)	44.5	41.4	14.1

p22phox (*CYBA*, rs4673)*			
Event Rate (%)	36.9	15.1	8.0
Number of patients	65	53	25
Number of patients (%)	45.5	37.1	17.5

TSP-4 (*THBS4*, rs1866389)*			
Event Rate (%)	17.9	36.6	50.0
Number of patients	95	41	4
Number of patients (%)	67.9	29.3	2.9

CETP (CETP, rs708272)*			
Event Rate (%)	14.9	26.4	33.3
Number of patients	47	72	24
Number of patients (%)	32.9	50.4	16.8

* Reported previously [12].

### Risk Models

To investigate potential associations of the PAI-2 polymorphism with recurrent coronary risk in the study group, Cox multivariable regression was performed in two stages as described in Methods. Initial entry into the model included all of the clinical parameters and biomarkers of Table 2 along with the polymorphisms of Table 3. Numbers of cases with complete data for all independent variables were 102 for the initial stage and 132 for the final stage. Results (Table 4) revealed significant and independent association of the PAI-2 polymorphisms with recurrent risk with highest risk for reference allele homozygotes. Further, each of the models demonstrated continued independent association with risk for the p22phox and TBS4 polymorphisms consistent with our earlier report while the CETP polymorphism lost significance [12].

**Table 4:** Results of Cox multivariable modeling.

Cox multivariable model	Hazard ratio	95% Confidence interval	p-value
Initial model (N=102)			
PAI-2 (rs6095)	0.38	0.16-0.90	0.027
p22phox (rs4673)	0.47	0.25-0.89	0.022
TBS4 (rs1866389)	2.50	1.26-4.93	0.008

Final model (N=132)			
PAI-2 (rs6095)	0.41	0.20-0.84	0.014
p22phox (rs4673)	0.44	0.24-0.79	0.006
TBS4 (rs1866389)	2.07	1.10-3.88	0.023

The initial stage of modeling consisted of a stepwise selection approach with inclusion of the seven clinical covariates (age, gender, race, prior MI, EF30, smoking, and BMI); seventeen blood biomarkers (apoB, cholesterol, Lp-PLA_**2**_, apoA-I, HDL-C, triglycerides, glucose, insulin, PAI-1, Lp(a), CRP, van Willebrand factor, fibrinogen, D-dimer, serum amyloid A, factor VII, and factor VIIa); and polymorphisms of PAI-2 (*SERPINB*, rs rs6095), LRP-1 (LRP1, rs1800156), p22phox (*CYBA*, rs4673), TBS4 (*THBS4*, rs1866389), and CETP (CETP, rs708272). The final stage of modeling consisted of an all-effects model that included significant parameters resulting from the initial stage of modeling (genetic polymorphisms of PAI-2, p22phox, and TBS4).

In order to assess robustness of the model with regard to clinical covariates traditionally associated with risk, clinical covariates (age, gender, race, prior MI, EF30, smoking, and BMI) were forced into the final model one-at-a-time. Additionally, potential effects of medication use were assessed in the same way by single-entry into the final model of medications (statins, beta blockers, aspirin, ACE inhibitors, calcium channel blockers, nitrates, and warfarin). In every case, both for the clinical covariates and medications, the three polymorphisms of the final model remained significant with hazard ratios essentially unchanged.

**Figure 2:**
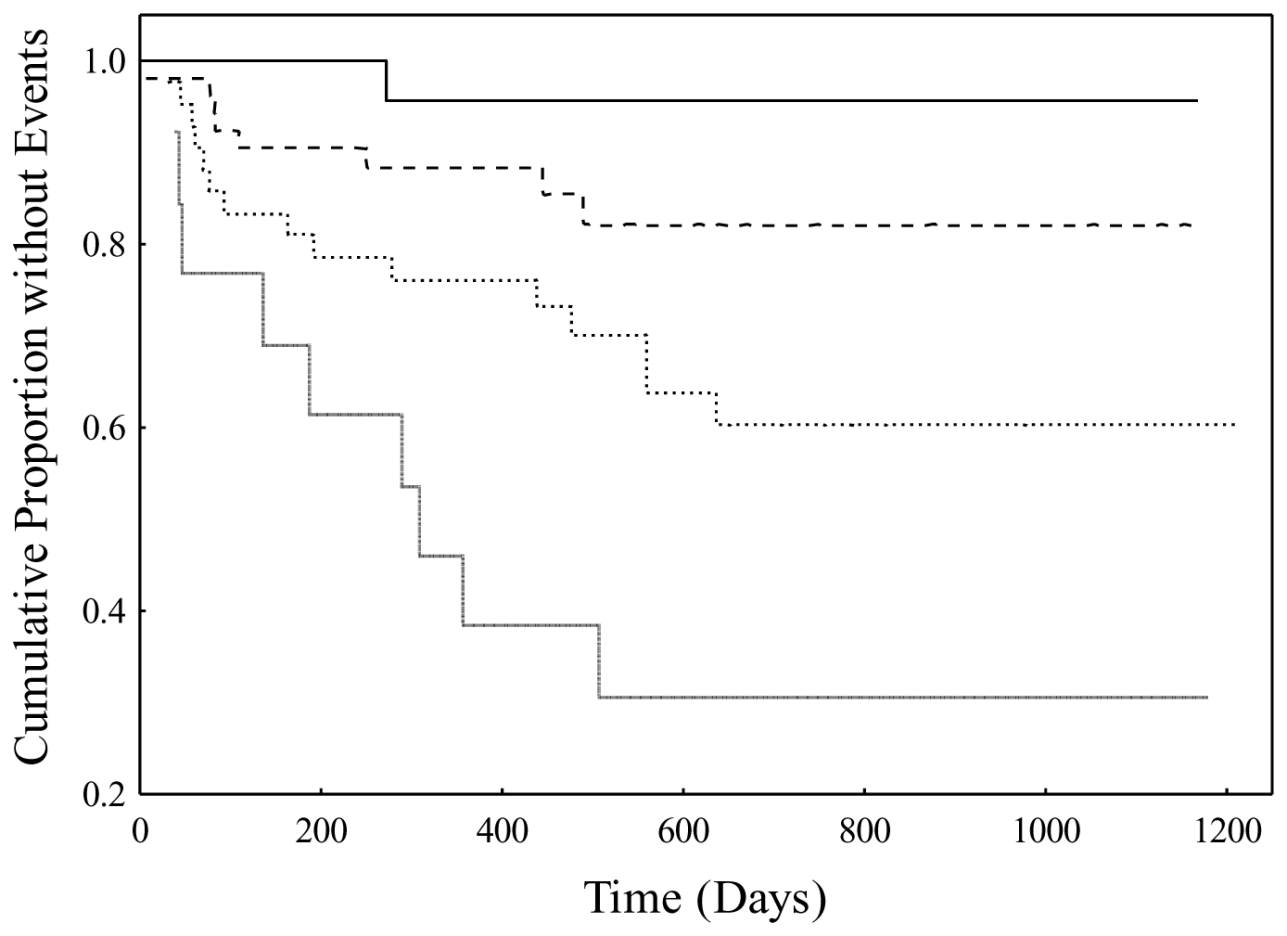
Kaplan-Meier plots in high HDL-C/high CRP high-risk subgroup as a function of the number of high-risk variants of dichotomized polymorphisms of PAI-2, p22phox, and TSP-4. Plots for patients were as follows: solid line - no high-risk variants (N=25), dashed line - any one high-risk variant (N=52), coarse dots - any two high-risk variants (N=42), and fine dots - all three high-risk variants (N=13).

To illustrate the effect in patients of having increasing numbers of high risk variants of the three significant polymorphism predictors of risk, outcome event rates were calculated as a function of numbers of high-risk variants present in patients. Results for outcome rates (%) and N’ s were as follows: no high-risk variants - 4.0%, N=25, any one high-risk variant - 15.4%, N=52, any two high-risk variants - 35.7%, N=42, and all three high-risk variants - 69.2%, N=13. Beyond the no high-risk variant group, there appeared to be an approximate doubling of the outcome rate with each additional high-risk variant. Figure 2 shows corresponding Kaplan-Meier plots.

### Interaction of LRP-1 and PAI-2 Polymorphisms

**Figure 3:**
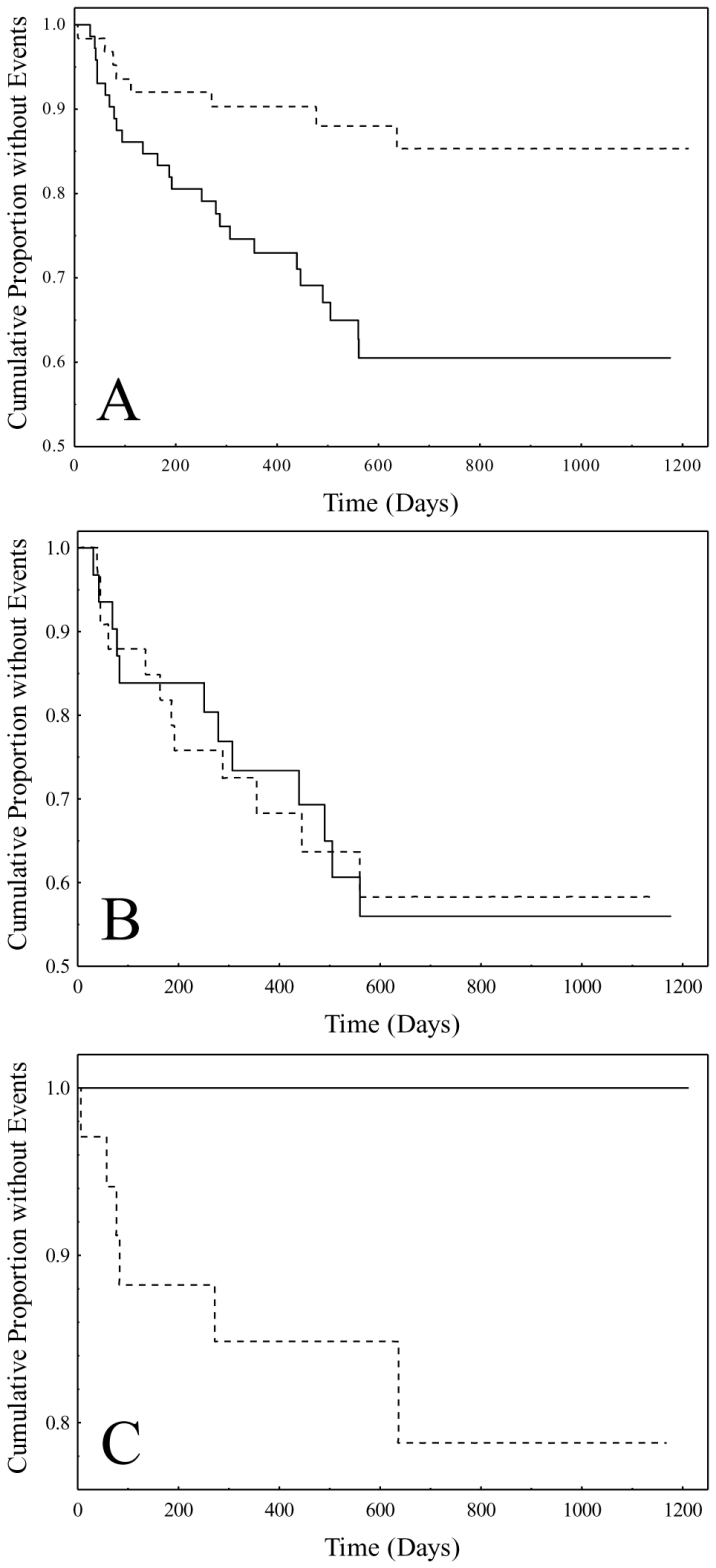
Kaplan-Meier analysis in high HDL-C/high CRP high-risk subgroup for PAI-2 (rs6095) and LRP-1 (rs1800156) polymorphisms. For the LRP-1 dichotomized polymorphism, curves were not different (p=0.34, plot not shown). (A) Kaplan-Meier curves for the dichotomized PAI-2 polymorphism: solid line - reference allele homozygotes, and dashed line - variant allele carriers (p=0.003). (B) Kaplan-Meier curves for reference allele homozygotes of the PAI-2 SNP further stratified according to the dichotomized LRP1 SNP: solid line - reference allele homozygotes of the LRP1 SNP (N=31), and dashed line - variant allele carriers of the LRP1 SNP (N=33); and (C) Kaplan-Meier curves for variant allele carriers of the PAI-2 SNP further stratified according to the dichotomized LRP1 SNP: solid line - reference allele homozygotes of the LRP1 SNP (N=23), and dashed line - variant allele carriers of the LRP1 SNP (N=34).

As noted above, formation of plasminogen activator/activator-inhibitor complexes with subsequent intracellular internalization via LRP-1 could play a role in PAI-mediated inhibition of fibrinolysis. To investigate potential interactive effects of the LRP-1 polymorphism on observed risk associations with the PAI-2 polymorphism, an interaction term between the LRP-1 and PAI-2 polymorphisms (dichotomized as reference allele homozygotes versus variant allele carriers) was added to the final Cox model of Table 4. Results demonstrated statistical significance for the interaction term (p=0.046). Kaplan-Meier analysis was then performed for combinations of the dichotomized PAI-2 and LRP-1 polymorphisms to illustrate interactive effects (Figure 3). Thus, for reference, Figure 3A shows curves for the PAI-2 SNP dichotomized as reference allele homozygotes (upper curve) and variant allele carriers (lower curve) which when compared were significantly different (p=0.003). Figure 3B shows the lower curve of Figure 3A stratified according to the LRP-1 polymorphism dichotomized as reference allele homozygotes (N=31) and variant allele carriers (N=33). The curves were not different from each other (p=0.99). Figure 3C shows the upper curve of Figure 3A similarly stratified by the dichotomized LRP-1 polymorphism according to reference allele homozygotes (N=23) and variant allele carriers (N=34). By contrast, the curves were significantly different (p=0.029) which was suggestive of an effect by the LRP-1 polymorphism on PAI-2 polymorphism-associated risk. Furthermore, the curve in Figure 3C corresponding to reference allele homozygotes of the LRP-1 polymorphism demonstrated absence of outcome events, a result presumably indicative of a protective effect against recurrent events in this patient group.

### Risk Discrimination of Models

To assess potential improvement in predictive ability over traditional risk models as related to findings of the current study regarding the PAI-2 polymorphism and its interaction with the LRP-1 polymorphism, discrimination abilities for two Cox proportional hazards models were evaluated. The first model was a reference model consisting of traditional clinical and blood biomarker levels as well as two polymorphisms previously demonstrated to be associated with risk in the current study population [12]. Thus, the reference model included age, gender, race, prior MI, smoking, BMI, apoB level, HDL-C level, and polymorphisms of p22phox and thrombospondin-4. The second model consisted of the reference model plus the PAI2 polymorphism and an interaction term between it and the LRP-1 polymorphism. Model discrimination was then assessed based upon the overall c index of Harrell, a statistic that is analogous to the area-under-the-curve (AUC) model discrimination statistic of ROC analysis [32,33]. For the reference model, the value of c was 0.7513; while for the second model, the value of c was 0.8028. These results were consistent with better predictive ability for the second model. 

## Discussion

The current study was an investigation assessing a potential role for fibrinolysis, the break-down of fibrin clot, in the establishment of recurrent coronary event risk in a subgroup of postinfarction patients previously defined as having high levels of HDL-C and CRP and to be at high-risk for recurrent events [11,12]. To this end, the study focused on a genetic polymorphism of the plasminogen-activator inhibitor, PAI-2. Results of the study demonstrated independent association of the PAI-2 polymorphism reference allele with recurrent CVD risk. In addition to PAI-2 polymorphism associated risk, results of the current study also demonstrated continued independent risk associations for polymorphisms of p22phox (*CYBA*) and TSP-4 (*THBS4*) as previously reported [12]. Findings were derived from stepwise Cox multivariable modeling that included simultaneous entry of clinical covariates and blood biomarkers as well as polymorphisms of PAI-2, LRP-1, p22phox, TSP-4, and CETP. In addition, further analyses were performed oriented toward identifying a potential link between the PAI-2 polymorphism and LRP-1 in view of the reported involvement of LDL endocytosis receptors in the regulation of PAI-mediated inhibition of fibrinolysis through internalization of plasminogen activator/plasminogen activator-inhibitor complexes. Results revealed interaction of the LRP-1 polymorphism on PAI-2 polymorphism-associated risk in that while LRP-1 polymorphism status had little effect on the higher-risk PAI-2 polymorphism variants, it split the lower-risk PAI-2 variants into two groups with significantly different outcome rates one of which (approximately 20% of study group patients) appeared to be protected against recurrent risk as evidenced by zero outcome events in the group.

More specifically, influence of the LRP-1 polymorphism on PAI-2 polymorphism-associated risk was illustrated using Kaplan-Meier analysis. Results showed higher-risk reference allele homozygotes of the PAI-2 polymorphism (N=64) unaffected by the LRP-1 polymorphism while lower-risk variant allele carriers of the PAI-2 polymorphism were split by the dichotomized LRP-1 polymorphism into lower- and higher-risk populations with the lower-risk population showing zero outcome events. The lower-risk population (N=23) was associated with reference allele homozygotes of the LRP-1 polymorphism while the higher-risk population (N=34) was associated with variant allele carriers of the LRP-1 polymorphism. This was consistent with a previous report showing association of risk for MI and coronary heart disease with variant allele carriers of the LRP-1 polymorphism [26]. As noted earlier, examination of effects of the LRP-polymorphism on the PAI-2 polymorphisms were prompted by reports of significant involvement of LRP-1 in regulation of fibrinolysis specifically in terms of its role in clearing plasminogen activator/plasminogen activator-inhibitor complexes from cell surfaces [20,21]. Thus, for example, uPA binds to cell surfaces via uPA receptors (uPAR) and in cases where local concentrations of extracellular PAI-2 are high as in inflammatory foci, PAI-2 forms complexes with uPA bound to uPAR that results in inactivation of uPA activity [20,21]. Such complexes can then interact with LRP-1 via the uPA moiety resulting in complex internalization but without the subsequent cell signaling events characteristic of internalization of corresponding complexes with PAI-1 [20,21]. It should be noted that after complex internalization, plasminogen activators and activator inhibitors are degraded while uPARs are recycled to the cell surface [21,34,35]. Recycling of unoccupied uPARs to the cell surface is thought to be critical for continued function regarding the generation of plasmin, and as such, it is considered to constitute a major action in the regulation of fibrinolysis [35]. In view of this sequence of events, it is clear that effects of LRP-1 polymorphism variants on PAI-2 action might influence fibrinolytic processes and serve as a basis for the differential CVD risk associations seen in the current study.

It should be noted that there is the potential for clinical application of current study findings as related to the high HDL-C/high CRP high-risk patient study group deriving from the risk model (PAI-2, p22phox, and TSP-4 gene polymorphisms) and the demonstrated interaction of the PAI-2 and LRP-1 polymorphisms. First, in terms of the risk model, there was the finding of an approximately doubling of outcome event rates with the presence of each additional polymorphism high-risk variant (zero high-risk variants -4.0%, any one high-risk variant -15.4%, any two high-risk variants -35.7%, and all three high-risk variants -69.2%). Thus genotyping of these polymorphisms in this patient group could add substantially to the ability to predict recurrent risk. Second, the finding of interaction of the PAI-2 and LRP-1 polymorphisms would also add to predictive ability of the model in terms of the splitting of the low-risk variant of the PAI-2 SNP by the LRP1 polymorphism resulting in a relatively high-risk group and a group with very low risk, the recognition of which could be of great value in terms of future medical management.

### Study Limitations

There were limitations in the current work that constrained the extent of study conclusions. No direct evidence was provided to support the presumptive major role of inflammation in the dysfunctional transformation of HDL particles nor was there any direct evidence regarding physico-chemical characterization of such particles. Relating to the PAI-2 and LRP-1 polymorphisms, in spite of the clear demonstration of associations with recurrent risk, causality for the establishment of risk with regard to the polymorphisms cannot be inferred from these statistical approaches. Along the same lines, there were no data provided in connection with actual biochemical functionality of these polymorphisms as related to fibrinolysis nor was there much to add in this regard from previous studies. It should also be noted at this point that although neither the PAI-2 polymorphism (intronic) nor the LRP-1 polymorphism (silent) would affect sequences of the respective proteins, potential functional effects could still be derived resulting from transcriptional and/or translational regulatory effects. Further constraints on study conclusions centered on limitations in patient numbers especially regarding the number of patient cases with complete data for all variables. However, in support of the validity of results of risk models was the finding that results were essentially unchanged when comparing results from the initial stepwise regression models that had fewer cases to final all-effect models with more cases. Additional limitations involved lack of potentially important data on clinical covariates (for example; diet, ethanol, exercise, social support, and mental status), blood biomarkers, and genetic polymorphisms of additional genes associated with fibrinolytic processes.

## Summary

The aim of the current study was to determine whether fibrinolysis plays a role in the development of risk in a group of postinfarction patients previously demonstrated to be at high-risk for recurrent coronary events and characterized by concurrently high levels of HDL-C and CRP. The main finding consistent with this notion was demonstration in the study group of independent association of recurrent risk with a genetic polymorphism of *SERPINB*; since PAI-2, its gene product, is thought to play a significant role in controlling fibrinolysis through inhibition of plasminogen activators especially in inflammatory foci. Furthermore, the finding of modulation of PAI-2 polymorphism associated risk by the LRP-1 polymorphism was consistent with a role for altered fibrinolysis in the development of risk in the study group. This follows from the central role of LRP-1 in the control of fibrinolysis through its mediation of cellular internalization of plasminogen activator/activator-inhibitor complexes. Indeed, it might be the case that the apparent protective effects in the patient subgroup manifesting zero outcome events could in some part be connected with interactions between the two polymorphisms in terms of resulting alterations in associated functionalities. Future studies should be oriented toward replicating study findings in independent populations both in terms of incident as well as recurrent coronary disease risk; and thereafter, assessing relative significance of alterations in fibrinolytic processes and underlying mechanistic pathways contributing to formation of risk in incident as well as recurrent coronary disease risk.
